# Downregulation of EZH2 in Trophoblasts Induces Decidual M1 Macrophage Polarization: a Potential Cause of Recurrent Spontaneous Abortion

**DOI:** 10.1007/s43032-021-00790-1

**Published:** 2021-11-24

**Authors:** Ye Shang, Shujuan Wu, SaiJiao Li, Xiaolin Qin, Jiao Chen, Jinli Ding, Jing Yang

**Affiliations:** 1 Reproductive Medicine Center, Renmin Hospital of Wuhan University, 238 Jie Fang Road, Wuhan, Hubei 430060 People’s Republic of China; 2 Hubei Clinic Research Center for Assisted Reproductive Technology and Embryonic Development, Wuhan, 430060 People’s Republic of China

**Keywords:** Decidual macrophages, Trophoblast, Enhancer of zeste homolog 2 (EZH2), Polarization, Immune regulation

## Abstract

Macrophages are known to be pivotal for ensuring the establishment of the immune tolerance microenvironment at the maternal–fetal interface. In particular, trophoblasts stay in close contact with decidual macrophages (DMs), which have been reported to play an active role in the modulation of the polarization of DMs. Thus, any dysfunction of trophoblasts might be associated with certain pregnancy‐related complications, such as recurrent spontaneous abortion (RSA). Enhancer of zeste homolog 2 (EZH2) is an important epigenetic regulatory gene that has been previously shown to be related to immune regulation. The present study assessed the expression of EZH2 in villi tissue obtained from healthy controls and RSA patients. Trophoblasts conditioned medium was collected to incubate macrophages differentiated from the THP‐1 cell line. The expression and function of EZH2 in trophoblasts were knocked down either by the use of siRNA or GSK126 as an inhibitor. Our results show a significant decrease in the expression of EZH2 in villi tissue from RSA patients as compared to healthy controls. Further, the inhibition of expression or function of EZH2 in trophoblasts promoted M1 macrophage polarization, which might be involved in the pathogenesis of RSA. Moreover, the suppression of EZH2 was found to affect the secretion of immune and inflammatory cytokines in trophoblasts. Altogether, these results indicated the importance of EZH2 in the regulation of immune functions of trophoblasts and thus highlighted its potential to be explored as a therapeutic target to prevent and treat pregnancy loss.

## Introduction

The success of a pregnancy requires the establishment of tolerance towards the semi‐allogeneic fetus and maintenance of host defense against pathogens, which are tightly associated with the coordinated balance between immune rejection and immune tolerance present at the maternal–fetal interface. In particular, the invading trophoblasts, decidual cells, endothelial cells, infiltrating immune cells, and cytokines secreted by the cells constitute an immune tolerance microenvironment that protects the fetus against rejection and attack [22]. Any disturbance in this immune balance has been shown to be associated with pregnancy‐related complications, such as pre‐eclampsia, premature delivery, and recurrent spontaneous abortion (RSA)[2].

During the first trimester of pregnancy, decidual macrophages (DMs) represent the second most abundant leukocytes at the maternal–fetal interface, accounting for 20–30% of decidual leukocytes [3, 6]. Importantly, DMs play important roles in immune modulation, immune‐suppressive activity, clearance of apoptotic cells, and spiral artery remodeling, which are essential for the establishment and maintenance of normal pregnancy [17]. Generally, macrophages are divided into two broad categories, classically activated (M1) and alternatively activated (M2) phenotypes. M1 are known to be proinflammatory and microbicidal in function and exhibit a high expression of CD80, CD86, INOS, IL‐23, and IL‐12. In comparison to this, M2 is immunomodulatory in function and is involved in ensuring tolerance and resolution of inflammation. M2 is characterized by the presence of CD163, CD206, CD209, and IL‐10 [7, 12, 14]. Macrophages exhibit a high degree of plasticity, and tissue macrophages can change their functional phenotype based on the surrounding microenvironment [19]. Interestingly, the polarization of DMs between M1 and M2 phenotypes has been observed throughout pregnancy. In particular, the M1 phenotype predominates during the preimplantation period and changes to the M2 phenotype following trophoblast attachment and invasion. Following this, it reverts to the M1 phenotype at the time of delivery. Thus, any aberration in the phenotype of DMs can have a detrimental effect on pregnancy outcomes. It might result in complications like pre‐eclampsia, preterm labor, intrauterine growth restriction, and RSA [13, 28]. Therefore, exploration of factors involved in the regulation of polarization of DMs at the maternal–fetal interface holds great significance.

In particular, trophoblasts represent the first point of contact between the blastocyst and maternal decidua. These trophoblasts play an active role in shaping the immunological milieu at the implantation site [22]. Besides this, DMs are found close to the trophoblast at the placenta [23]. Several studies have shown that certain factors secreted by trophoblasts regulate the polarization of DMs [1, 37]. Thus, any dysfunction of the trophoblast might lead to inappropriate polarization of DMs and might be involved in the pathogenesis of pregnancy complications.

Enhancer of zeste homolog 2 (EZH2) is the core catalytic subunit of polycomb repressive complexes 2 (PRC2), which mediates the transcriptional silencing of target genes via H3K27me3 [4, 30]. Aberrant expression of EZH2 has been reported in multiple tumors. Several pieces of evidences suggested its role in the regulation of immune cell function [36]. Additionally, EZH2 plays an important role in reproduction. A significantly decreased expression of EZH2 was reported in the villi of RSA women [18]. Moreover, it was previously shown that EZH2 suppression in glioblastoma shifts microglia towards the M1 phenotype, and the knockdown of EZH2 inhibited the expression of anti‐inflammatory factors while promoting the expression of pro‐inflammatory factors in glioblastoma cells [34]. Thus, it was hypothesized that the expression of EZH2 in trophoblast might affect the polarization of DMs and participate in the pathogenesis of RSA.

The present study reported a decreased expression of EZH2 in villi tissue obtained from RSA patients. Downregulation of EZH2 in trophoblasts affected the polarization of macrophages, inducing an elevation in M1‐associated markers and reduction in M2‐associated markers. Furthermore, EZH2 suppression in trophoblasts affected the secretion of immune and inflammatory cytokines. Thus, the results of this study indicated a regulatory effect of trophoblasts on the polarization of macrophages, and the suppression of EZH2 in trophoblasts disturbed the immune regulatory function. These findings highlighted the potential of EZH2 to be explored as an immunotherapeutic target to prevent RSA.

## Materials and Methods

### Patient and Samples

We enrolled 6 healthy women (who underwent elective termination for unwanted pregnancy at 6–12 weeks of gestation) and 5 patients with RSA (gestation ages were between 6 and 10 weeks) at the Renmin Hospital of Wuhan University (Wuhan, China) between September 2020 and June 2021. The exclusion criteria for the subjects were as follows: (a) endocrine or metabolic disease, (b) karyotype abnormalities, and (c) uterine abnormality. RSA was defined as the sequential loss of two or more pregnancies before 20 weeks of pregnancy. The gestation ages for healthy controls and RSA patients were 8.67 ± 1.63 and 9.40 ± 1.14 weeks, respectively. The samples were collected following the informed consent from all patients. The human placental villous tissues were fixed in 4% paraformaldehyde for paraffin embedding in blocks, and the remaining were frozen in liquid nitrogen and stored at − 80℃. All performances were approved by the Review and Ethics Boards of Renmin Hospital of the Wuhan University.

### Cell Culture and Treatments

The trophoblast cell line HTR-8/SVneo (HTR-8) was obtained from the China Center for Type Culture Collection (Wuhan, China) and grown in DMEM/F-12 medium (Gibco) supplemented with 10% fetal bovine serum (FBS; Gibco), 100 U/mL penicillin, and 100 mg/mL streptomycin (Sigma-Aldrich). In addition, human monocyte cell line THP-1 was obtained from the Institute of Biochemistry and Cell Biology, Chinese Academy of Sciences (Shanghai, China) and cultured in RPMI-1640 medium supplemented with 10% FBS, 100 U/mL of penicillin, and 100 mg/mL of streptomycin. The cells were cultured at 37℃ under a humidified atmosphere with 5% CO_2_.

EZH2 siRNA (si-EZH2, sense: GGAUGGUACUUUCAUUGAATT and antisense: UUCAAUGAAAGUACCAUCCAG) and negative control (si-NC) were purchased from WZ Biosciences. HTR-8 cells were seeded in 6-well plates (4 × 10^5^ cells/well) 24 h before the transfection, and siRNA (80 nM) was transfected with Lipofectamine 3000 (Invitrogen) according to the manufacturer’s instructions. After 24 h of the transfection, the cells were cultured in DMEM/F12 media with 1% FBS for 48 h. The conditioned medium (CM) was collected and stored at − 80℃ until further use. HTR-8 cells were treated with EZH2 functional inhibitor GSK126 (Sigma-Aldrich) or dimethyl sulfoxide (DMSO, Sigma-Aldrich) at a concentration of 10 μM for 48 h, after which the CM was collected and stored as mentioned earlier.

For macrophage polarization, THP-1 cells were differentiated into M0 macrophages by treating with 50 ng/mL of phorbol 12-myristate 13-acetate (PMA; Sigma-Aldrich) for 24 h. M0 macrophages were then incubated in CM (at 33% concentration) for 48 h.

### Flow Cytometry

The cells were collected to prepare a cell suspension and washed twice with phosphate-buffered saline (PBS). The cells were then incubated with PerCP/Cyanine5.5 anti-human CD86 (Biolegend), and PE anti-human CD163 (Biolegend) for 30 min at 4℃. After the washing step, the cells were analyzed by the Beckman Coulter Cytoflex Flow cytometer, and the data obtained were analyzed with the FlowJo software (Version 10.6.2). The mean fluorescence intensity (MFI) was used to represent the expression level of each antigen in the macrophages.

### Quantitative Reverse Transcription-Polymerase Chain Reaction (qRT-PCR)

Total RNA was extracted using the RNAex Pro Reagent (Accurate Biotechnology) according to the manufacturer’s instructions. In addition, RNA was reverse-transcribed according to the mRNA Reverse Transcription Kit (Accurate Biotechnology); qPCR was performed using the SYBR Green Premix Pro Taq HS qPCR Kit (Accurate Biotechnology) on a 7500 RT PCR System (Applied Biosystems, Foster City, CA, USA). GAPDH was selected as an internal control, and all qPCR reactions were performed in triplicate. The relative mRNA expression level was calculated using the 2^−ΔΔCt^ method. The specific primers were listed in Table 1.

**Table 1:** Specific primers

Gene	Primer sequence (5′ to 3′)	Size (bp)
EZH2	F:GGACGAAGAATAATCATGGGCC	116
	R:CGTCTGAACCTCTTGAGCTGTCT	
GAPDH	F:AGAAGGCTGGGGCTCATTTG	258
	R:AGGGGCCATCCACAGTCTTC	
IL-10	F:TCAAGGCGCATGTGAACTCC	176
	R:GATGTCAAACTCACTCATGGCT	
TGF-β	F:GACTCGCCAGAGTGGTTATCT	154
	R:CGGTAGTGAACCCGTTGAT	
IL-1β	F:AGCTACGAATCTCCGACCAC	186
	R:CGTTATCCCATGTGTCGAAGAA	
TNF-α	F: TCTCGAACCCCGAGTGACAA	181
	R: TGAAGAGGACCTGGGAGTAG	
IL-6	F:AATAACCACCCCTGACCCAAC	149
	R:ACATTTGCCGAAGAGCCCT	
IL-4	F:CGGCAACTTTGTCCACGGA	111
	R:TCTGTTACGGTCAACTCGGTG	
CXCL-16	F:GACATGCTTACTCGGGGATTG	170
	R:GGACAGTGATCCTACTGGGAG	
PD-L1	F:GGACAAGCAGTGACCATCAAG	235
	R:CCCAGAATTACCAAGTGAGTCCT	

### Western Blotting

Total protein was extracted from cells or tissues by using the ice-cold radioimmunoprecipitation assay (RIPA, Servicebio) containing phosphatase repressor (Servicebio), cocktail (Servicebio), and phenylmethanesulfonyl fluoride (PMSF, Servicebio) and centrifuged at 12,000 × *g* for 20 min at 4℃. The protein concentration was measured with BCA solution (Beyotime). About 30 μg of the protein was electrophoresed in SDS–polyacrylamide gels and transferred onto a PVDF membrane. This membrane was then incubated with 5% non-fat milk for 1 h at room temperature and subsequently incubated with a primary antibody against EZH2 (1:1000; Cell Signaling Technology) and GAPDH (1:5000; Proteintech) overnight at 4℃. The blots were washed thrice with Tris-buffered saline and 0.1% Tween20 (TBST) and then incubated with HRP-conjugated secondary antibody (Proteintech) for 1 h at room temperature. After the final washing step, the blots were analyzed by using the Chemiluminescence Western Detection System (Bio-Rad, Hercules, CA, USA).

### Immunohistochemistry (IHC)

The paraffin-embedded villi tissues were cut into sections then deparaffinized, rehydrated in water, and washed in PBS three times. Next, we used 3% hydrogen peroxide to block the endogenous peroxidase activity. Non-specific binding was blocked with bovine serum albumin for 20 min. The sections were then incubated with a primary antibody against EZH2 (1:50) at 37 ℃ overnight and then incubated with a secondary antibody for 30 min. Antibodies binding were detected with a brown precipitate, followed by staining with 3,3-diaminobenzidine (DAB) (Dako Cytomation, Glostrup, Denmark). Finally, the sections were counterstained with hematoxylin and dehydrated with 95% alcohol.

### Statistical Analysis

Statistical analyses were performed using SPSS 25.0. Student’s t-tests were used to analyze the statistical significance of the differences. The difference was considered to be statistically significant at *p* < 0.05, and all the data were expressed as mean ± standard deviation (SD).

## Results

### Decreased Expression of EZH2 in Villi Tissue in RSA Patients

**Fig. 1:**
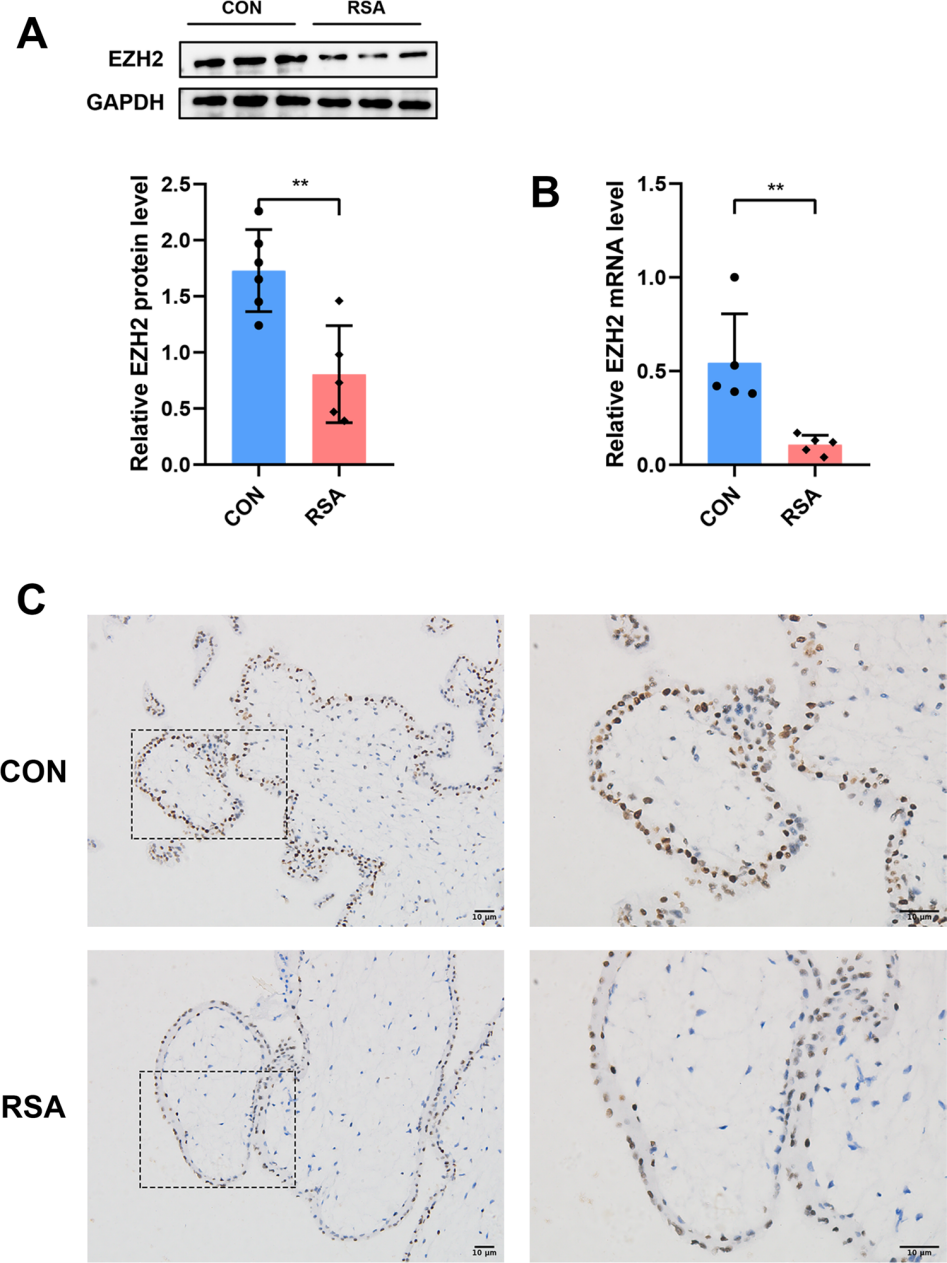
The expression of EZH2 was decreased in the villi tissues from RSA patients. **A** The EZH2 protein levels in the villi tissues from healthy controls (*n* = 6) and RSA patients (*n* = 5) were determined by Western blotting. **B** The relative expression of EZH2 mRNA in villi tissues from healthy controls (*n* = 5) and RSA patients (*n* = 5). **C** Immunohistochemical staining of EZH2 in the villi tissues from healthy controls and RSA patients. Data represented mean ± SD, **p < 0.01; scale bar = 10 μm

To assess the expression of EZH2 in clinical samples, RT‐PCR and western blot analyses were conducted to evaluate mRNA expression and protein levels of EZH2, respectively, in villous tissues obtained from RSA patients and healthy controls. The results showed that both mRNA expression and protein levels of EZH2 were significantly lower in villi tissue obtained from RSA patients as compared to healthy controls (Fig. 1A and B). Besides this, IHC staining of serial sections showed that EZH2 was mainly located in cytotrophoblast, and a decreased expression of EZH2 was observed in villi tissue obtained from RSA patients (Fig. 1C). These results were consistent with the findings of a previous study [18].

### EZH2 Suppression in Trophoblasts Promoted M1 Polarization of Macrophages

**Fig. 2:**
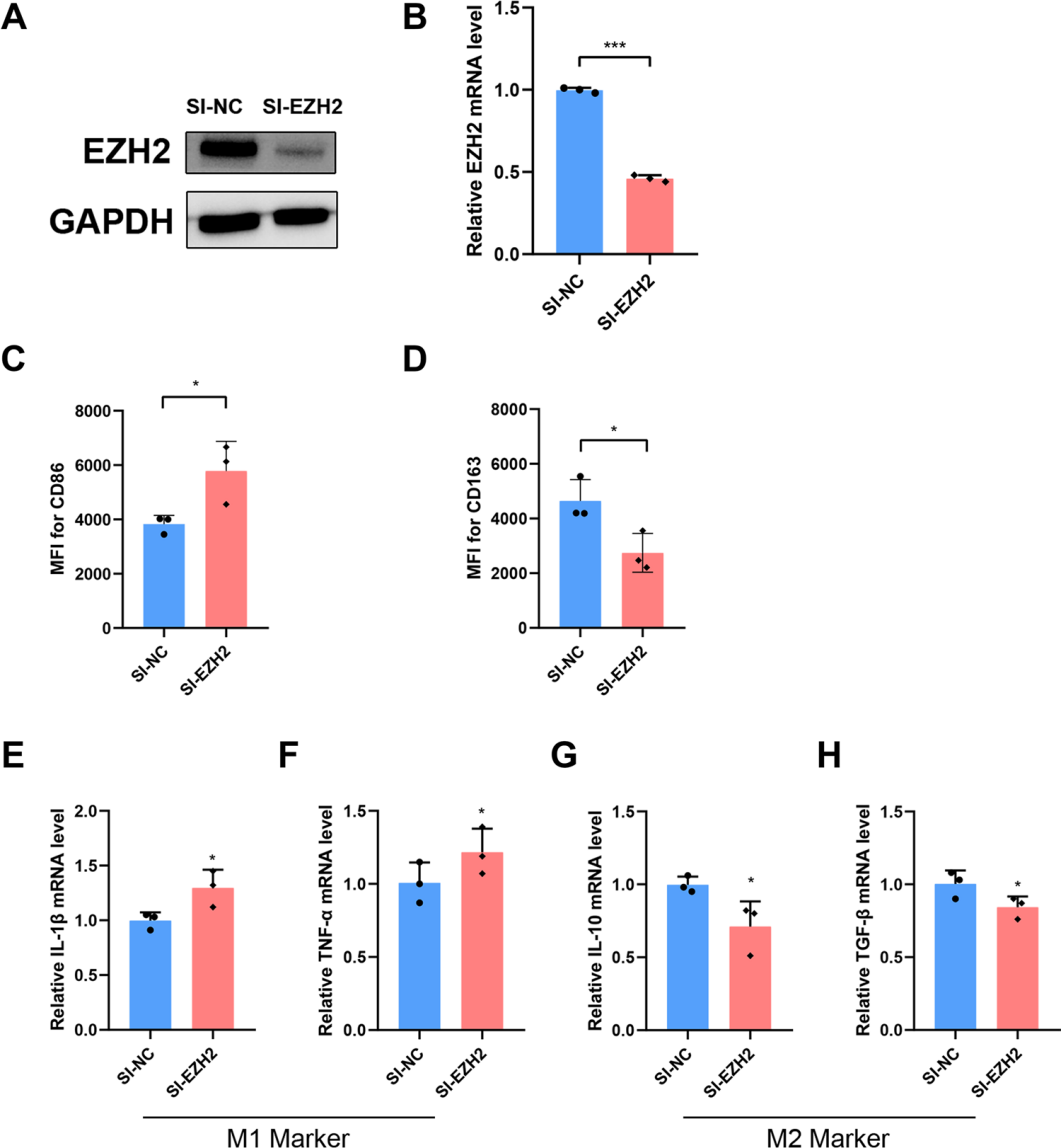
EZH2 knocked down in trophoblasts promoted M1 macrophage polarization. **A** The EZH2 protein levels in HTR-8 cells transfected with si-EZH2 (80 nM) or si-NC after 48 h. **B** The relative expression of EZH2 mRNA in the HTR-8 cells transfected with si-EZH2 (80 nM) or si-NC after 48 h. **C** and **D** Macrophages differentiated from THP-1 cells treated with different CM were analyzed by flow cytometry to measure the MFI of CD86 and CD163. **E**–**H** Relative expression of IL-1β, TNF-α, IL-10, and TGF-β mRNA in macrophages differentiated from THP-1 cells. Data represented mean ± SD, *p < 0.05, **p < 0.01

The results for the previous study conducted in our laboratory and several other studies have confirmed that trophoblasts exhibit the ability to induce M2 phenotype polarization without any physical contact [1, 5, 37]. In order to explore the influence of EZH2 on macrophage polarization, the expression of EZH2 in human trophoblast cell line HTR‐8 was first knocked down via transfection with EZH2 specific siRNA. As shown in Fig. 2A and B, both mRNA expression and protein levels of EZH2 were significantly reduced in HTR‐8 cells. Following this, macrophages differentiated from the THP‐1 cell line were incubated with CM of HTR‐8 cells. The results showed that macrophages treated with CM, collected from si‐EZH2-transfected HTR‐8 cells (si‐EZH2 group), exhibited a significantly increased MFI of CD86 (Fig. 2C), but a lower MFI of CD163 was observed (Fig. 2D). Moreover, macrophages in the si‐EZH2 group displayed a higher expression of M1 markers (IL‐1β and TNF‐α) and lower levels of M2 markers (IL‐10 and TGF‐β) (Fig. 2E–H).

**Fig. 3:**
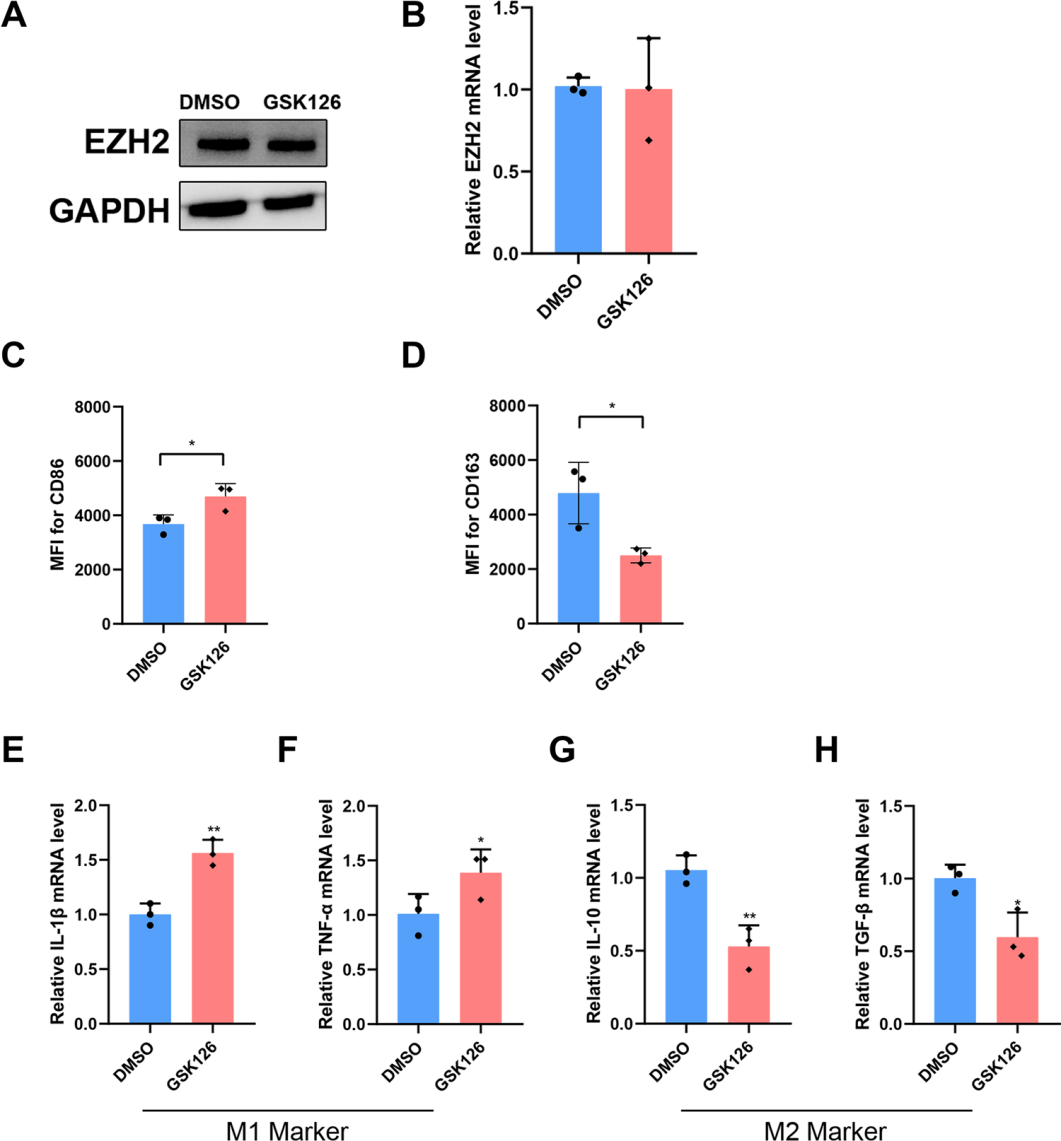
The inhibition of EZH2 function in trophoblast-promoted M1 macrophage polarization. **A** The EZH2 protein levels in HTR-8 cells treated with GSK126 (10 nM) or DMSO after 48 h. **B** The relative expression of EZH2 mRNA in HTR-8 cells treated with GSK126 (10 nM) or DMSO after 48 h. **C** and **D** Macrophages differentiated from THP-1 cells treated with different CM were analyzed by flow cytometry to measure the MFI of CD86 and CD163. **E**–**H** The relative expression of IL-1β, TNF-α, IL-10, and TGF-β mRNA in macrophages differentiated from THP-1 cells. Data represented mean ± SD, *p < 0.05, **p < 0.01

Next, the effect of inhibition of EZH2 function in trophoblasts on macrophage polarization was assessed. In particular, EZH2 inhibitor GSK126 was selected to inhibit the function of EZH2. GSK126 treatment did not affect the expression of EZH2 in HTR‐8 cells (Fig. 2A and B). In the GSK126 group (macrophages treated with CM collected from HTR‐8 cells pre‐treated with GSK126), the MFI of CD86 was found to be increased (Fig. 3C), whereas the MFI of CD163 was significantly reduced (Fig. 3D). Meanwhile, mRNA expression levels of IL‐1β and TNF‐α were found to be increased (Fig. 3E and F), while mRNA expression of IL‐10 and TGF‐β was decreased (Fig. 3G and H). These results were in concordance with the results obtained for the si‐EZH2 group.

These results further suggested that inhibition of EZH2 expression and function in trophoblasts could affect its immune regulatory function, promoting M1 macrophage polarization.

### EZH2 Modulates the Expression of Immune and Inflammatory Factors in Trophoblasts

**Fig. 4:**
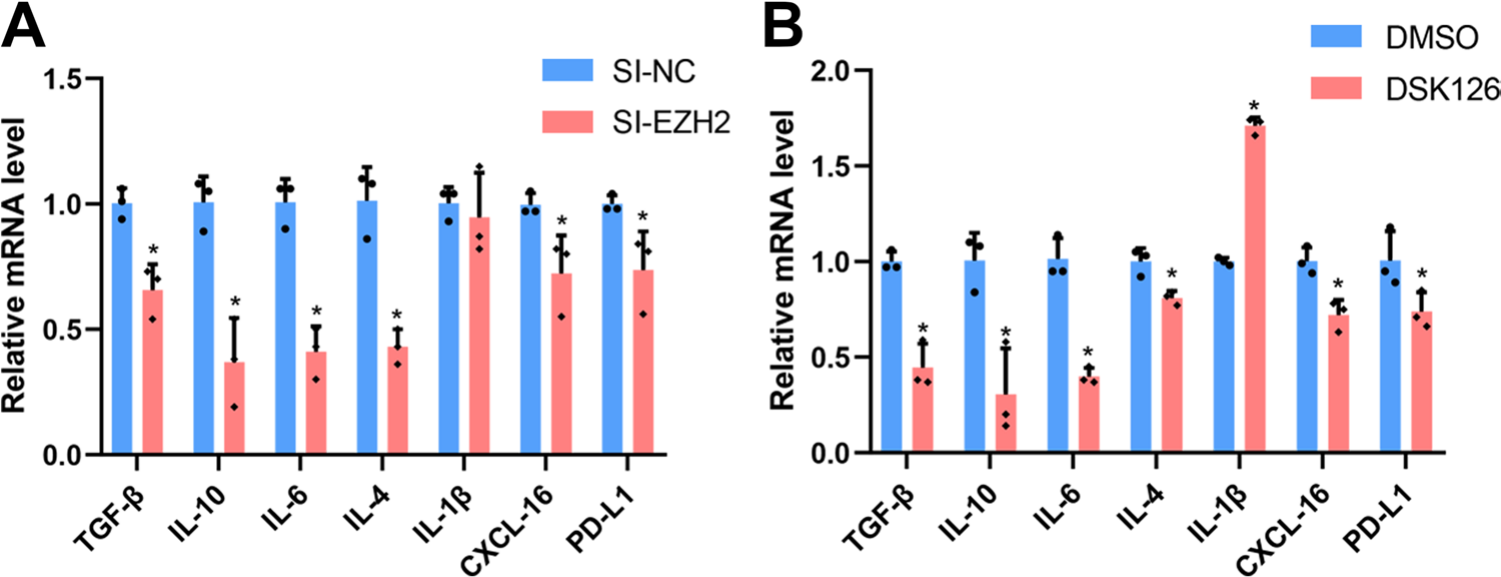
EZH2 suppression affected the expression of immune and inflammatory cytokines in trophoblasts. **A** HTR-8 cells were transfected with si-EZH2 (80 nM) or si-NC for 48 h, and the relative mRNA levels of TGF-β, IL-10, IL-6, IL-4, IL-1β, CXCL-16, and PD-L1 were determined by qPCR. **B** HTR-8 cells were treated with GSK126 (10 nM) or DMSO for 48 h, and the relative mRNA levels of TGF-β, IL-10, IL-6, IL-4, IL-1β, CXCL-16, and PD-L1 were determined by qPCR. Data represented mean ± SD, *p < 0.05

In order to investigate the potential mechanism involved in EZH2-mediated modulation of immune regulation in trophoblasts, correlational researches were considered, and it was found that EZH2 might be associated with the expression of genes related to immune function and inflammation [29, 34, 36]. Thus, the expression of immune and inflammatory cytokines related to DMs polarization was assessed in trophoblasts. The results indicated that silencing of EZH2 in HTR‐8 cells decreased the expression of TGF‐β, IL‐10, IL‐6, IL‐4, CXCL‐16, and PD‐L1 (Fig. 4A). Additionally, inhibition of EZH2 function via GSK126 showed similar changes in terms of cytokine levels. In particular, mRNA expression levels of TGF‐β, IL‐10, IL‐6, IL‐4, CXCL‐16, and PD‐L1 were reduced, whereas IL‐1β levels were found to be elevated (Fig. 4B). These results suggested that EZH2 might be involved in the regulation of DMs polarization via regulation of the expression of immune and inflammatory cytokines in trophoblasts.

## Discussion

Pregnancy is a complicated physiological process that involves the formation of a fetus and associated appendages and systematic adaptation of the maternal organs. During pregnancy, the maternal immune system undergoes dramatic changes as gestation processes [27]. Macrophages are one of the main leukocyte populations found at the maternal–fetal interface, and unique macrophage phenotypes and heterogeneity play an important role in the establishment and maintenance of a successful pregnancy [38]. DMs exhibit varied phenotypes during different stages of pregnancy [27]. During the preimplantation period, M1 activation is induced in DMs [11]. As trophoblasts attach to the endometrium, macrophages switch to a mixed M1/M2 profile, and this mixed polarization pattern exists throughout the first trimester. When the placentation and remodeling of spiral arteries get completed, DMs predominantly shift towards M2 phenotype to prevent rejection of fetus and allow fetal growth until parturition [31]. Although it has been a topic of argument that DMs belong neither to M1 nor M2 subsets [10], some studies have suggested that M2 macrophages or M2 subgroup constitute the predominant phenotype at decidua [9, 27]. However, a decline in the percentage of M2 and an increase in the proportion of M1 might lead to adverse pregnancy outcomes, such as preterm birth, pre‐eclampsia, fetal growth restriction, and RSA[38].

The environment in which macrophages mature and differentiate is important for the polarization of macrophages. And the polarization of DMs during pregnancy was regulated and influenced by the cells at the maternal–fetal interface involving trophoblasts, decidual stromal cells, and other immune cells through various cytokines and immune checkpoints [27]. In particular, DMs are found in close proximity to trophoblasts at the maternal–fetal interface. It has been previously reported that trophoblasts play an active role in shaping the immunological milieu during pregnancy [17]. Thus, it is expected that surrounding trophoblasts might play an important role in the regulation of polarization of DMs. It has been previously shown that conditioned media from first trimester trophoblasts could induce differentiation of monocytes into a unique macrophage phenotype and express immunoregulatory genes that are representative of M2‐like macrophage [1]. In particular, trophoblast-secreted factors, including M‐CSF, IL‐10 [26], IL‐34 [16], IL‐6 [5], IL‐8, TGF‐β [1], CXCL‐16 [33], PD‐L1 [37], galectin‐9 [15], hyaluronan [32], receptor activator for nuclear factor‐κB ligand (RANKL) [21], and vasoactive intestinal peptide [25], have been reported to be involved in the induction of M2‐like polarization of macrophages at decidua. Thus, any dysfunction of trophoblasts might alter cytokine secretion profile, resulting in inappropriate polarization of DMs.

Epigenetic regulation has emerged as one of the key mechanisms involved in controlling proper gene expression [20]. EZH2, the catalytic subunit of PRC2, mediates the methylation of H3K27 and is linked to the silencing of gene expression [34, 36]. The role of EZH2 has been extensively studied in oncology, and its importance in reproduction has also been verified [24]. It has been previously reported that EZH2 played an essential role in the development of early mouse preimplantation embryos, mediated via regulation of epigenetic modification and apoptosis [8]. Additionally, EZH2 was identified as a novel driver of EMT in endometriosis [35]. Besides this, Lv et al. reported that EZH2 might regulate trophoblast invasion as an epigenetic factor, and downregulation of EZH2 attenuated trophoblast invasion that was involved in the pathogenesis of RSA [18]. In the present study, a decreased expression of EZH2 was observed in villi tissue obtained from RSA patients, which was consistent with the findings of a previous study [18]. In recent years, several studies demonstrated the importance of EZH2 in the regulation of immune cell functions and inflammation. The suppression of EZH2 in glioblastoma was shown to be associated with immune response, which induced changes in the secretion of immune cytokines [34]. Thus, it was conjectured that decreased expression of EZH2 in trophoblasts might be involved in the abnormal polarization of DMs. In the present study, both the inhibition of expression and function of EZH2 in trophoblasts resulted in a decreased expression of M2‐associated markers and increased the expression of M1‐associated markers. The expression of CD86 was found to be significantly increased, while the expression of CD163 declined. The expression of EZH2 in trophoblasts disturbed the phenotypic differentiation of macrophages. In addition to this, the results of the present study also suggested that suppression of EZH2 altered the secretion of immune and inflammatory cytokines in trophoblasts. In particular, the levels of TGF‐β, IL‐10, IL‐6, IL‐4, CXCL‐16, and PD‐L1 declined, which have been previously reported to be associated with the polarization of M2 macrophages at decidua. Comparatively, the expression of IL‐1β was found to be increased. However, the mechanism involved in EZH2-mediated regulation of secretion of immune and inflammatory cytokines in trophoblasts needs to be clarified in future studies. Additionally, trophoblast‐secreted cytokines related to the polarization of DMs need to be further studied.

Altogether, the present study demonstrated that downregulation of EZH2 in trophoblasts induced polarization of M1 macrophages, which might be mediated via modulation of secretion of immune and inflammatory cytokines in trophoblast. The decreased expression of EZH2 in trophoblast could possibly influence the microenvironment present at the maternal–fetal interface, leading to inappropriate polarization of DMs. Therefore, the study highlighted the potential of EZH2 to be explored as a therapeutic target to prevent and treat pregnancy loss.
